# LincRNA-p21 promotes mesenchymal stem cell migration capacity and survival through hypoxic preconditioning

**DOI:** 10.1186/s13287-018-1031-x

**Published:** 2018-10-25

**Authors:** Shan-Shan Meng, Xiu-Ping Xu, Wei Chang, Zhong-Hua Lu, Li-Li Huang, Jing-Yuan Xu, Ling Liu, Hai-Bo Qiu, Yi Yang, Feng-Mei Guo

**Affiliations:** Department of Critical Care Medicine, Zhongda Hospital, School of Medicine, Southeast University, No.87, Dingjiaqiao Road, Gulou District, Nanjing, 210009 China

**Keywords:** LincRNA-p21, Mesenchymal stem cell, Migration, Hypoxic preconditioning, Acute respiratory distress syndrome

## Abstract

**Background:**

Mesenchymal stem cells (MSCs) derived from bone marrow have potent stabilizing effects for the treatment of acute respiratory distress syndrome (ARDS). However, low efficiency and survival in MSC homing to injured lung tissue remains to be solved. Therefore, the aim of this study was to assess whether large intergenic noncoding RNA (LincRNA)-p21 promote MSC migration and survival capacity through hypoxic preconditioning in vitro.

**Methods:**

MSCs were cultured and divided into the normoxia culture group (20% O2) and hypoxia culture group (1% O2). To determine roles and mechanisms, lentivirus vector-mediated LincRNA-p21 knockdown of MSCs and hypoxia-inducible factor (HIF-1α) inhibitor KC7F2 were introduced. Additionally, MSC migration was analyzed by scratch test and transwell migration assays. MSC proliferation was tested by cell counting kit-8 and trypan blue dye. Apoptosis was detected by Annexin V-PE/7-AAD stained flow cytometry. Moreover, LincRNA-p21 and HIF-1α mRNA was measured by reverse transcription-polymerase chain reaction, and HIF-1α and CXCR4/7 protein were assayed by western blot (WB) or enzyme-linked immunosorbent assay (ELISA). Apoptosis protein caspase-3 and cleaved-caspase-3 were investigated by WB analysis. Considering interactions between VHL and HIF-1α under LincRNA-p21 effect, co-immunoprecipitation was detected.

**Results:**

Hypoxic preconditioning MSC promoted migration capacity and MSC survival than normoxia culture group. MSCs induced by hypoxic preconditioning evoked an increase in expression of LincRNA-p21, HIF-1α, and CXCR4/7(both were chemokine stromal-derived factor-1(SDF-1) receptors). Contrarily, blockade of LincRNA-p21 by shRNA and HIF-1α inhibitor KC7F2 abrogated upregulation of hypoxic preconditioning induced CXCR4/7 in MSCs, cell migration, and survival. Furthermore, co-immunoprecipitation assay revealed that hypoxic preconditioning isolated VHL and HIF-1α protein by increasing HIF-1α expression.

**Conclusions:**

Hypoxic preconditioning was identified as a promoting factor of MSC migration and survival capacity. LincRNA-p21 promotes MSC migration and survival capacity through HIF-1α/CXCR4 and CXCR7 pathway under hypoxic preconditioning in vitro.

## Background

Acute respiratory distress syndrome (ARDS) is characterized by pulmonary edema and diffuse inflammation [1, 2]. To date, despite gradually improved mechanical ventilation, there are no effective and approved treatments for ARDS [1, 2], remaining a high mortality rate of 40% [3]. In recent years, mesenchymal stem cell (MSC) derived from bone marrow-based cell therapy is a potential and ideal therapeutic approach for ARDS. MSCs are widely used in the study of stem cells therapy for ARDS because of their capacity of target homing, self-renewal, multipotent differentiation, and paracrine productions. MSC homing to damaged lung tissue is a key process for ARDS treatment. Despite reported success, it is demonstrated that the amount of engrafted MSCs homing to target lung tissues was lower than 5% [4, 5]. Thus, a new strategy for MSC migration is needed.

Recent studies have shown that cells may be preconditioned by exposure to selected stresses to induce prior expression of cytoprotective genes [6], such as hypoxia, small-molecule pharmacological agents, oxidative stress, heat shock, cytokines, growth factors, or biophysical stimuli [7, 8]. Hypoxic preconditioning MSC is known to be extremely protective against subsequent lethal hypoxia damage [9, 10]. Recent studies have also shown that short-term exposure of MSCs to hypoxia can enhance cell survival properties and boost the tissue repair capability [11, 12]. Hypoxic preconditioning has been extensively studied in a number of organs and tissues in relation to diseases to enhance therapeutic effects, such as cardiomyocytes [11, 13–18], endocrine [19], intestinal tract [20], kidney [21, 22], and liver [23].

Long non-coding RNAs (LncRNAs) are characterized with RNA molecules larger than 200 nt with no protein-coding capability [24]. LncRNAs take part in the regulation of cell growth and movement. They regulate transcription via chromatin modulation, post-transcriptional regulation, organization of protein complexes, and allosteric regulation of proteins [25]. The large intergenic non-coding RNA-p21 (LincRNA-p21), located in chromosome [26], was first described in 2010. Recently, LincRNA-p21 has been reported that it is involved in the contribution of hypoxia to the Warburg effect in cancer cells [27]. LincRNA-p21 was induced by HIF-1α under hypoxic conditions, which disrupts the HIF-1α-VHL interaction, thus upregulating the available HIF-1α and regulation of angiogenesis and the Warburg effect [27–31]. However, the relationship between LincRNA-p21 and MSC migration and survival capacity under hypoxic preconditioning in vitro were not certain.

The aim of this study was to determine whether LincRNA-p21 promoted MSC migration capacity through hypoxic preconditioning in vitro. We introduced hypoxic preconditioning on MSC in vitro and then explored LincRNA-p21/HIF-1α/CXCR4 and CXCR7 pathway to investigate cell migration and survival capacity.

## Methods

### Cell culture and protocol

MSCs derived from bone marrow of female C57BL/6 mouse were purchased from Cyagen Bioscience, Inc. (Guangzhou, China) and general identified according to surface phenotypes and multipotency by supplier. DMEM/F12 containing 10% fetal bovine serum growth medium (Wisent, Nanjing, China) and humidified 5% CO2 incubator at 37 °C were suitable for MSC cultivate. And culture media were changed every 2–3 days. MSCs of passages 3–7 from the same batch were finally used for all experiment.

MSCs were cultured and divided into the normoxia culture group (20% O2) and hypoxia culture group (1% O2) through inflating different nitrogen. Each group received different treatments for 0 to 24 h. Before some experiments, MSC were treated with HIF-1α inhibitor KC7F2 (20 μM, Selleck, USA). For Transwell migration assay, SDF-1 (100 ng/ml, Millipore, MA) were used.

### Lentivirus vector-mediated LincRNA-p21 knockdown in MSCs and EGFP reporter gene detection

MSCs of passages less than 6 were used for LincRNA-p21 knockdown experiment. The LincRNA-p21 knockdown was conducted using lentivirus vector (1) Target Seq: TAGCGAGAGCATTGACACTTA; (2) Target Seq: AGGGTTCTGTCTGCACCTCAT; (3) Target Seq: GACCAGAACTGGAGCCAACAA) and knockdown-specific for EF-1α-enhanced green fluorescent protein (EGFP) was used as a negative control. 293T cells were used to package the lentivirus with the aid of three packaging plasmids (one was vector plasmids carrying target sequences, two were virus packaging plasmids), and a higher titer of lentivirus was obtained for following experiments. MSCs were transfection and screened by antibiotic puromycin for 7–14 days. Subsequently, MSCs carrying empty vectors and EGFP (shRNA-control) or MSCs carrying both the LincRNA-p21 gene and EGFP (shLincRNA-p21) were harvested.

### Scratch test

We rowed five straight lines on the back of 30-mm petri dishes with 0.5–1 cm intervals. Add 2 ml (2.5 × 10^5^ cells/ml) MSC into petri dishes and cultivate for 24 h. Next scratch was made with 10-μl pipetting spear perpendicular to the five base lines. Then, wash cells with PBS and add 2-ml serum-free medium to observe cells.

### Transwell migration assay

Besides scratch test, MSC migration ability could also be tested by transwell chambers (24-well plate, 8-μm pore size). MSCs were suspended in serum-free medium and adjusted to a density of 1 × 10^5^ cells/ml. One hundred microliters of cell suspension was added to the upper chamber of the migration well. To the contrary, serum-free medium and chemokine stromal-derived factor-1(SDF-1, 100 ng/ml, Millipore) were loaded into the lower chamber. After a 24-h incubation with hypoxia or normoxia culture at 37 °C, the cells from the top of the filter were excluded with a cotton swab, and the cells that had migrated through to the underside of the insert membranes were fixed with 4% paraformaldehyde for 10 min and stained with crystal violet (Beyotime, Haimen, China) for 20 min. Cells in five random separate microscope fields were counted (400-fold magnification).

### Cell proliferation assays and apoptosis assays

To determine the effects of hypoxic preconditioning on the oxidative stress-induced proliferation and apoptosis of MSCs, MSCs were treatment with or without 250 μM H_2_O_2_ for 6 h in serum-free DMEM/F12. At the same time, MSCs were induced by normoxia and hypoxia stimulation for 6 h. Cell proliferation was evaluated by Cell Counting Kit-8 (CCK-8, Beyotime, Shanghai, China) and trypan blue dye. MSCs were seeded into 96-well plates with different stimulations. In CCK-8 analysis, CCK8 was added for 2 h and absorbance was tested using a micro-plate reader with 450-nm wavelengths. In trypan blue dye experiment, MSCs were digested by trypsinization and resuspended into cell suspension and were stained with 0.1% trypan blue at room temperature for 5 min. Cell suspension was counted on the cell counter plate and analysis for the ratio of living cells.

Cell apoptosis were analyzed by Annexin V-PE/7-AAD stained flow cytometry (BD Biosciences, USA). MSCs were harvested through trypsinization and washed with PBS and then centrifuged to collect the pellet. The pellet was resuspended in 1 × binding buffer at a density of 5.0 × 10^5^ cells per mL. One hundred microliters of the binding buffer was incubated with 5 μL of PE-conjugated Annexin V (BD) and 5 μL of 7-AAD (BD) and added to samples for 15 min at room temperature (25 °C) in the dark. Samples were analyzed by flow cytometer (ACEA NovoCyte, China) using NovoExpress (ACEA NovoCyte). Early apoptotic(PE positive, 7-AAD negative), late apoptotic, and dead cells (PE positive, 7-AAD positive) can be discriminated on the basis of a double-labeling for Annexin V-PE and 7-AAD and analyzed by flow cytometry.

### Real-time quantitative-PCR (RT-qPCR)

MSCs were collected and isolated RNA immediately. Total RNA was isolated with TriPure Isolation Reagent (Roch, Switzerland). And RNA concentration was determined with microplate reader (Infinite M200 Pro, Tecan, Switzerland). Reverse transcriptase was performed (Thermo Fisher Scientific, USA) for cDNA synthesis. Specific primer pairs were designed with the Primer Express software (Vector NTI advance10). The following primers were used: GAPDH (133 bp), sense 5′-ACAACTTTGGCATTGTGGAA-3′ and antisense 5′-GATGCAGGGATGATGTTCTG-3′; and LincRNA-p21 (122 bp), sense 5′-CCACATTGCTGTTCATCACC-3′ and antisense 5′-TGAGCAAGCTAGTGGAAGCA-3′; and HIF-1α (118 bp), sense 5′-CTCATGGAGGCCAGAAGAAG′ and antisense 5′-GGGCTAGTGAGATGGCTCAG′. Real-Time PCR System (Applied Biosystems, USA) was used to detect the PCR product caused by the binding of SYBR Green (Thermo Fisher Scientific) to dsDNA. The threshold cycle (CT) meant the number of PCR cycles that are required for the fluorescence signal to exceed the detection threshold value. The CT of each target product was associated with the amplification plot of GAPDH. The level of RNA from target gene was described as gene expression. Fold difference was calculated by the distinction of CT values between hypoxia culture group and normoxia culture group. The relative quantitative results showed changes of gene expressions in hypoxia culture samples when compared with those in normoxia culture ones.

### Western blot (WB) analysis

RIPA lysis buffer supplemented with 1 mmol/l phenylmethanesulfonyl fluoride (Beyotime) were used to extract total proteins of MSCs. Extract total proteins of MSCs were separated with SDS-PAGE condensed electrophoresis (Beyotime) and transferred onto polyvinylidene fluoride membranes (Beyotime). Then, we blocked the membranes in 5% BSA and dyed them for 1 h at room temperature and dyed at 4 °C overnight with primary antibodies against β-Actin (1:1000; Cell Signaling, USA), HIF-1α (1:1000;Cell Signaling), Hyp564 HIF-1α (1:1000;Cell Signaling), CXCR4 (1:100; Abcam, USA), CXCR7(1:1000;Abcam), Caspase-3 (1:1000;Cell Signaling), and Cleaved-Caspase-3 (1:1000;Cell Signaling). Peroxidase-conjugated secondary antibody (1:3000; Fcmacs) was used to incubate the membrane at room temperature for 1 h. Finally, ECL was applied to detect the bands with a chemiluminescence imaging system (Bioshine ChemiQ 4800mini, Ouxiang, Shanghai, China).

### Co-immunoprecipitation

We used co-immunoprecipitation method to identify proteins which might associate with interest protein. Cells lysed were obtained by treating with extraction buffer and sonicate and cleared by centrifuging at 14,000*g* for 10 min at 4 °C. Protein content was normalized with protein assay kit (Bio-Rad Laboratories, USA). The supernatant was incubated overnight at 4 °C with 1:250 dilutions of the primary antibody; protein A/G Agarose (Beyotime) was added and incubated for additional 4 h at 4 °C. After washing, the immune complexes were boiled in SDS sample buffer. These samples were subjected to immunoblotting with HIF-1α (1:50; Cell Signaling), VHL (1:50; Thermo Fisher Scientific) according to the manufacturer’s instructions, respectively.

### Enzyme-linked immunosorbent assay (ELISA)

Supernatants of MSCs with different stimulations were collected and centrifuged to remove debris. HIF-1α was tested according to the manufacturer’s instructions of HIF-1α EILSA sets (Elabscience, China). All samples were repeated three times.

### Statistical analyses

SPSS 19.0 and Graphpad Prism 7.0 software were used to analyze experimental data. The mean ± standard deviation was presented. Tukey’s multiple comparison tests and one-way analysis of variance were applied for group comparisons. Statistical significance was defined by *p* value < 0.05.

## Results

### Hypoxic preconditioning promotes MSC migration and survival

**Fig. 1:**
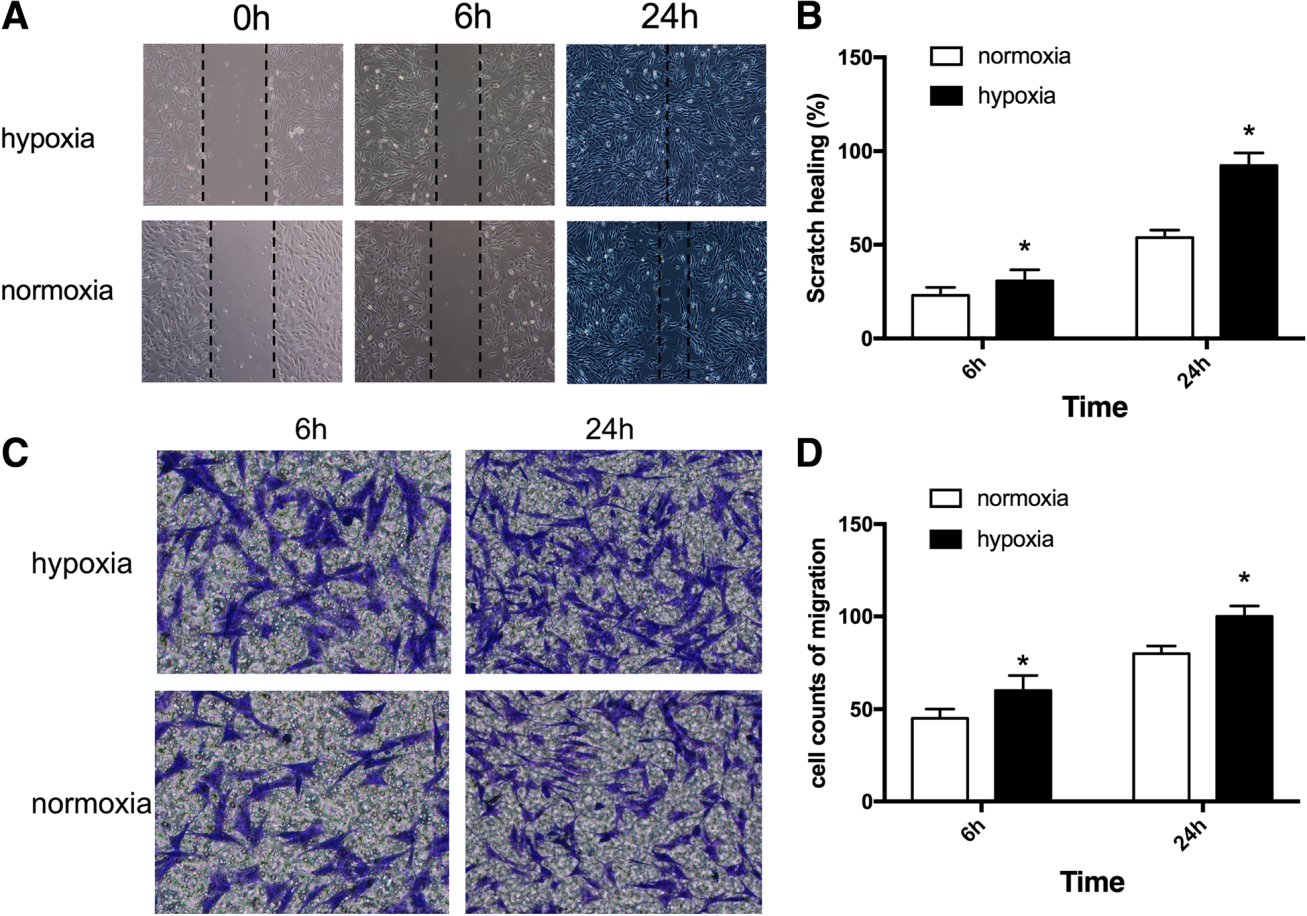
The effect of hypoxic preconditioning on MSC migration. Hypoxic preconditioning (1% O_2_)-induced MSCs migration were tested by scratch healing and transwell migration assays (stained with crystal violet). **a** The effect of hypoxic preconditioning on MSC migration in scratch healing analysis at 6 h and 24 h (400-fold magnification). **b** Healing area/wounded area ratio in scratch healing analysis at 6 h and 24 h. **c** The effect of hypoxic preconditioning on MSC transwell migration capacity stained with crystal violet at 6 h and 24 h (400-fold magnification). **d** Cell counts of transwell migration at 6 h and 24 h. Results are mean ± SD (*n* = 3). *p < 0.05 vs. group normoxia

The effects of hypoxic preconditioning on MSC migration were mimic in vitro. First, we evaluate MSC migration capacity by introducing scratch test to assess healing area/wounded area ratio with 6 h and 24 h hypoxia treatment. The figure (Fig. 1a, b) showed that hypoxic preconditioning promoted MSC healing area/wounded area after 6 h and 24 h scratch injury than normoxia culture group. Second, we used transwell migration assays and crystal violet staining to calculate migration cell numbers after 6 h and 24 h hypoxia. The results (Fig. 1c, d) illustrated that hypoxic preconditioning increased MSC migration numbers than normoxia culture group. The results revealed that hypoxic preconditioning raised MSC migration.

**Fig. 2:**
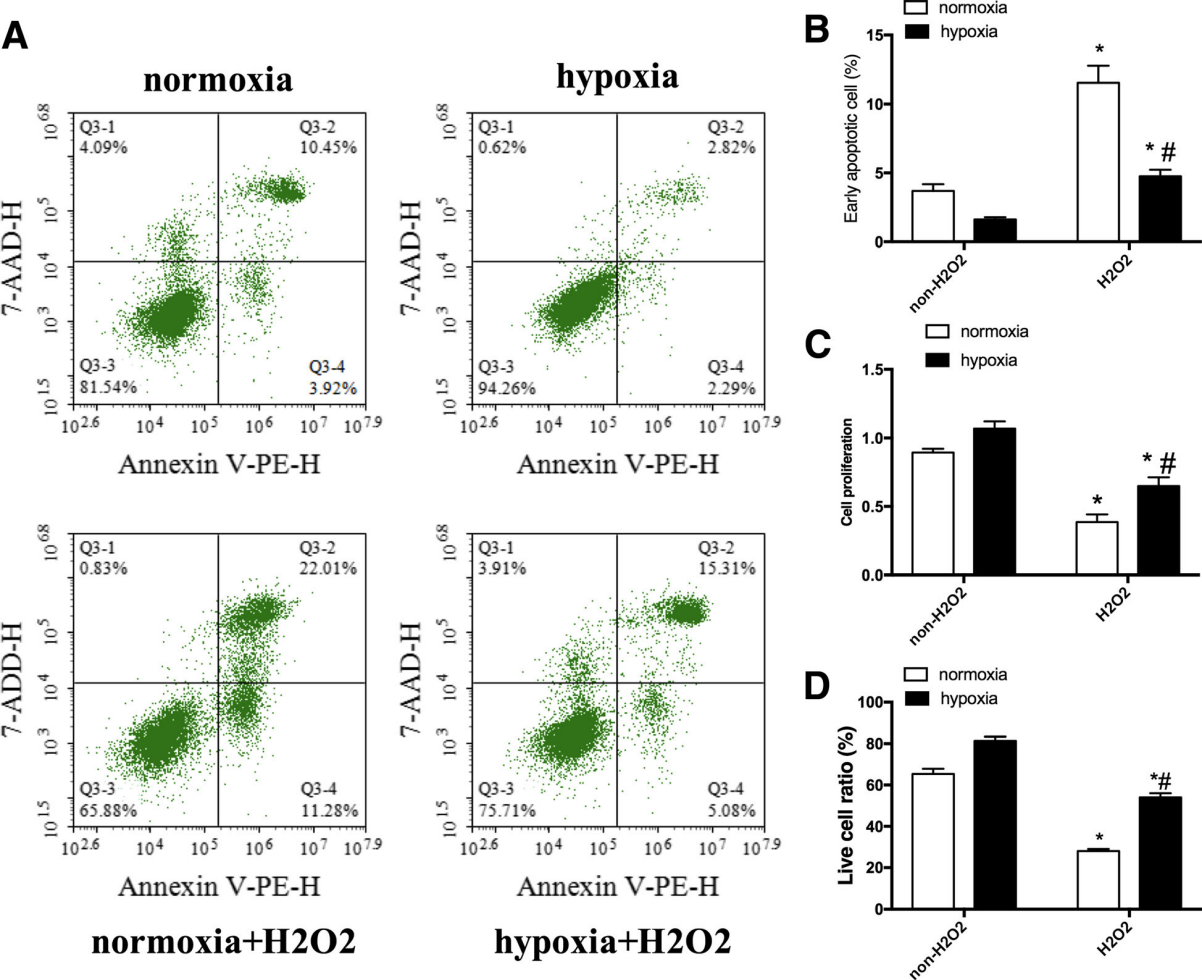
The effect of hypoxic preconditioning to MSC survival. Hypoxic preconditioning (1% O_2_)-induced MSC proliferation and apoptosis were tested by CCK8, trypan blue dye, and Annexin V-PE/7-AAD stained flow cytometry method. **a** The effect of hypoxic preconditioning on MSC apoptosis with Annexin V-PE/7-AAD stained flow cytometry method at 6 h. **b** Early apoptotic MSCS under hypoxic preconditioning at 6 h. **c** MSC proliferation under hypoxic preconditioning tested by CCK8 method at 6 h. **d** MSC proliferation under hypoxic preconditioning tested by trypan blue dye at 24 h. Results are mean ± SD (*n* = 3). *p < 0.05 vs. group non-H_2_O_2_; ^#^*p* < 0.05 vs. group normoxia

To determine the effects of hypoxic preconditioning on the oxidative stress-induced proliferation and apoptosis of MSCs, H_2_O_2_ (250 μM) were introduced. Hypoxic preconditioning enhanced MSCs survival by increasing cell proliferation and reducing cell apoptosis. The effect of hypoxic preconditioning on MSC apoptosis and proliferation were relatively further assessed using Annexin V-PE/7-AAD-stained flow cytometry, CCK8 reagent, and trypan blue dye to observe the survival of MSCs in 6-h hypoxic stimulation. The effects showed that H_2_O_2_ treatment increased the ratio of apoptosis cells (Fig. 2a, b) and decreased viability cells (Fig. 2c, d), and hypoxic preconditioning improved MSC proliferation and attenuate MSC apoptosis (Fig. 2) than the normoxia culture group. This implied that hypoxic stimulation had the effects of cell survival.

### Hypoxic preconditioning raises LincRNA-p21/HIF-1α and promotes upregulation of CXCR4/7 in MSCs

**Fig. 3:**
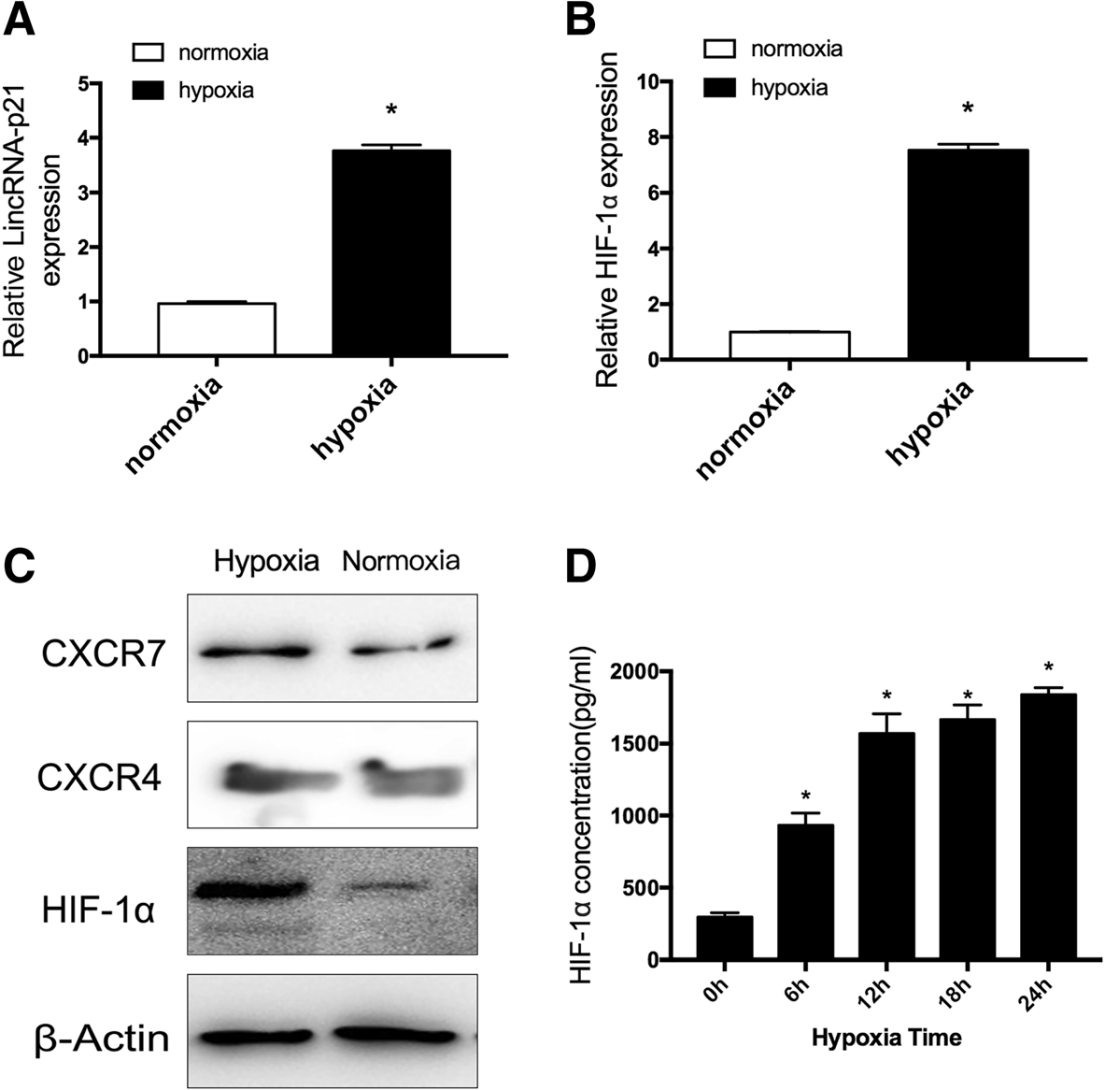
The effect of hypoxic preconditioning on MSC to LincRNA-p21, HIF-1α, and CXCR4/7. RT-PCR, WB analysis, and ELISA kits were applied to test hypoxic preconditioning (1% O_2_) induced MSCs signaling change at 24 h. **a** The effect of hypoxic preconditioning on MSC to LincRNA-p21 relative quantitative expression. **b** The effect of hypoxic preconditioning on MSC to HIF-1α mRNA relative quantitative expression. **c** The effect of hypoxic preconditioning on MSC to HIF-1α and CXCR4/7 protein expression using WB analysis. **d** HIF-1α concentration in hypoxic preconditioning induced MSC supernatant. Results are mean ± SD (*n* = 3). *p < 0.05 vs. group normoxia or hypoxia time 0 h

To assess whether LincRNA-p21, HIF-1α, and CXCR4/7 in MSCs after 24-h hypoxia preconditioning were involved in enhanced MSC migration and survival, nucleic acid and protein were abstracted with MSCs lysis for PCR or western blot analysis. RT-qPCR method (Fig. 3a, b) displayed that hypoxic preconditioning on MSC increased the expression of LincRNA-p21 and HIF-1α mRNA than the normoxia culture group. WB analysis (Fig. 3c) showed that hypoxic preconditioning on MSC also added HIF-1α and CXCR4/7 protein amount than normoxia culture group. As expected, HIF-1α concentration in cellular supernatant was also responded to higher in hypoxic preconditioning (Fig. 3d). Collectively, these data highlighted that hypoxic preconditioning on MSC increased the expression of LincRNA-p21, HIF-1α, and CXCR4/7.

### HIF-1α activates CXCR4/7 signaling by hypoxic preconditioning and promotes MSC migration and decreased MSC apoptosis

**Fig. 4:**
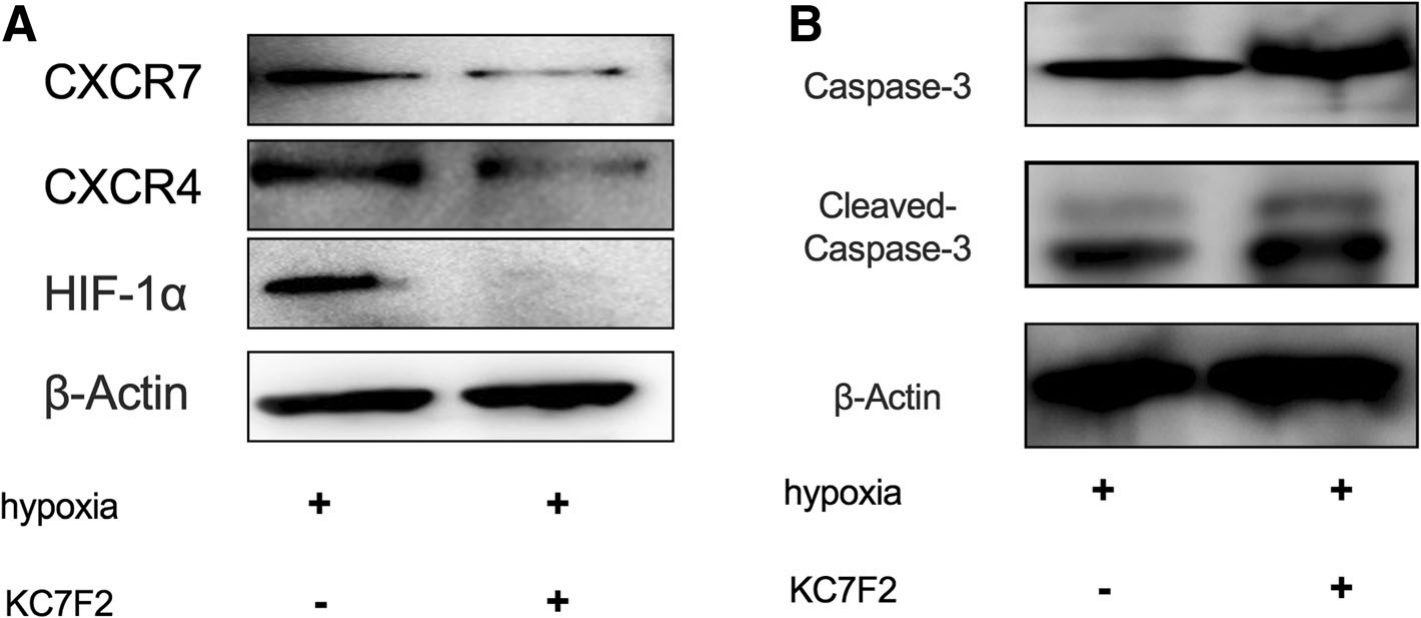
The effect of HIF-1α inhibitor KC7F2 on hypoxic preconditioning induced MSC. To investigate the role of HIF-1α inhibitor KC7F2 on hypoxic preconditioning induced MSC, we introduced KC7F2 (20 μM) for 24-h treatment and used WB analysis and immunofluorescence to test hypoxic preconditioning (1% O_2_)-induced MSCs CXCR4/7 and apoptosis caspase-3 change. **a** The effect of HIF-1α inhibitor KC7F2 on HIF-1α and CXCR4/7 protein expression with WB analysis in hypoxic preconditioning induced MSC. **b** The effect of HIF-1α inhibitor KC7F2 on apoptosis protein caspase-3 and cleaved-caspase-3 expression with WB analysis in hypoxic preconditioning induced MSC

To investigate the involvement of HIF-1α in the progress of hypoxic preconditioning on MSCs, the inhibitor KC7F2 was used. With 24-h inhibitor treatment, WB analysis revealed that KC7F2 inhibited the CXCR4 and CXCR7 upregulation in hypoxia preconditioning on MSC (Fig. 4a). Furthermore, in the presence of KC7F2, apoptosis-associated caspase-3 and cleaved-caspase-3 level was increased compared with the non-KC7F2 group tested by WB analysis (Fig. 4b). Taken together, these data confirmed that HIF-1α activated CXCR4/7 signaling by hypoxic preconditioning and promoted MSC migration and decreased MSC apoptosis.

### LincRNA-p21 contributes to HIF-1α-mediated expression of CXCR4/7 in MSC migration capacity and survival, and hypoxia-induced LincRNA-p21 stabilized HIF-1α- VHL interactions

**Fig. 5:**
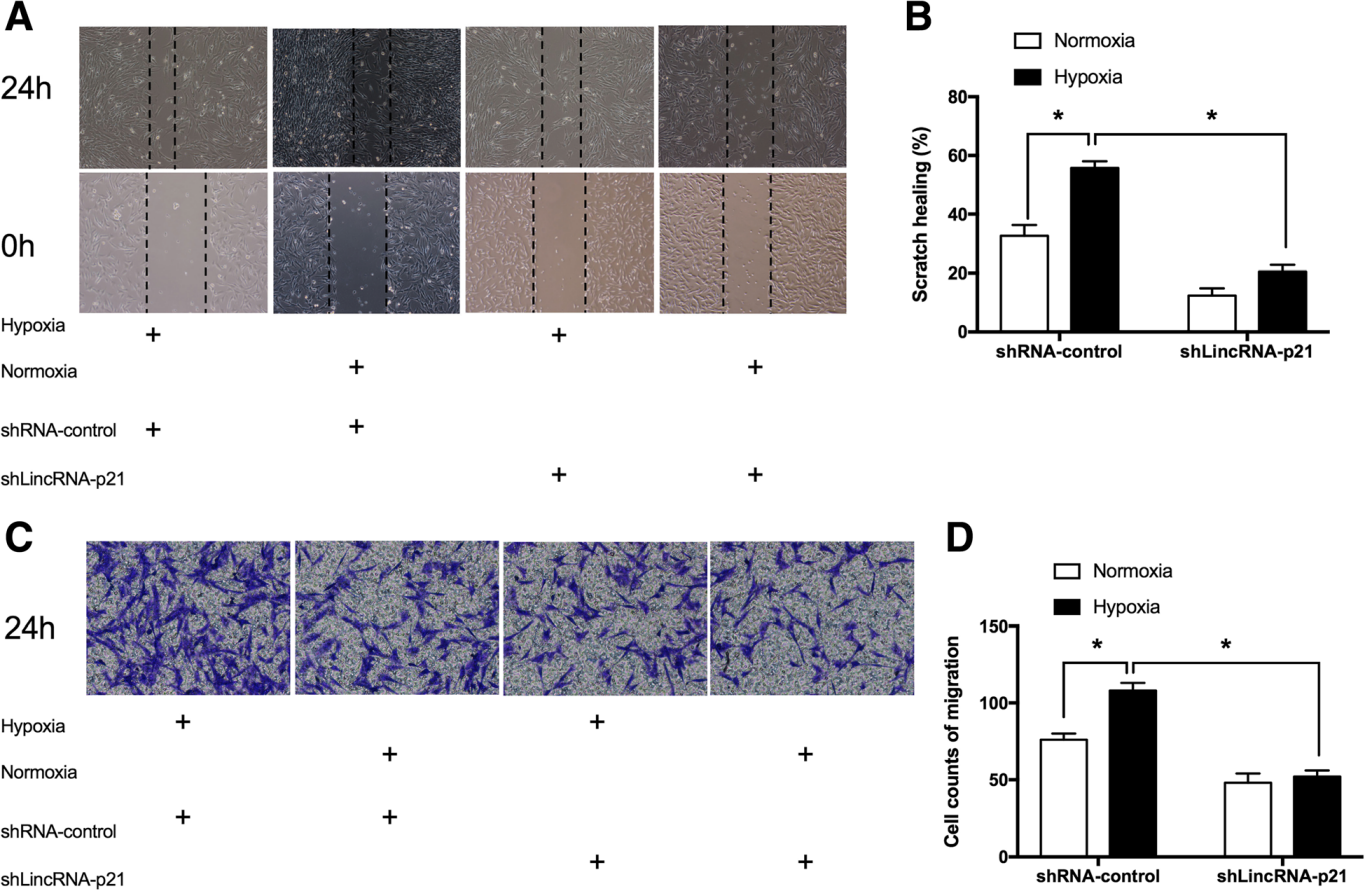
The effect of knock down of LincRNA-p21 on hypoxic preconditioning-induced MSC migration. To investigate the role of LincRNA-p21 on hypoxic preconditioning induced MSC migration, we introduced shLincRNA-p21 and used hypoxic preconditioning (1% O_2_) induced MSCs migration at 24 h, which were tested by scratch healing and transwell migration assays (stained with crystal violet). **a** The effect of knock down of LincRNA-p21 on hypoxic preconditioning-induced MSC migration in scratch healing analysis at 24 h (400-fold magnification). **b** Healing area ratio in scratch healing analysis at 24 h. **c** The effect of knock down of LincRNA-p21 on hypoxic preconditioning induced MSC transwell migration at 24 h (400-fold magnification). **d** Cell counts of transwell migration at 6 h and 12 h. Results are mean ± SD (*n* = 3). *p < 0.05 vs. comparison group

**Fig. 6:**
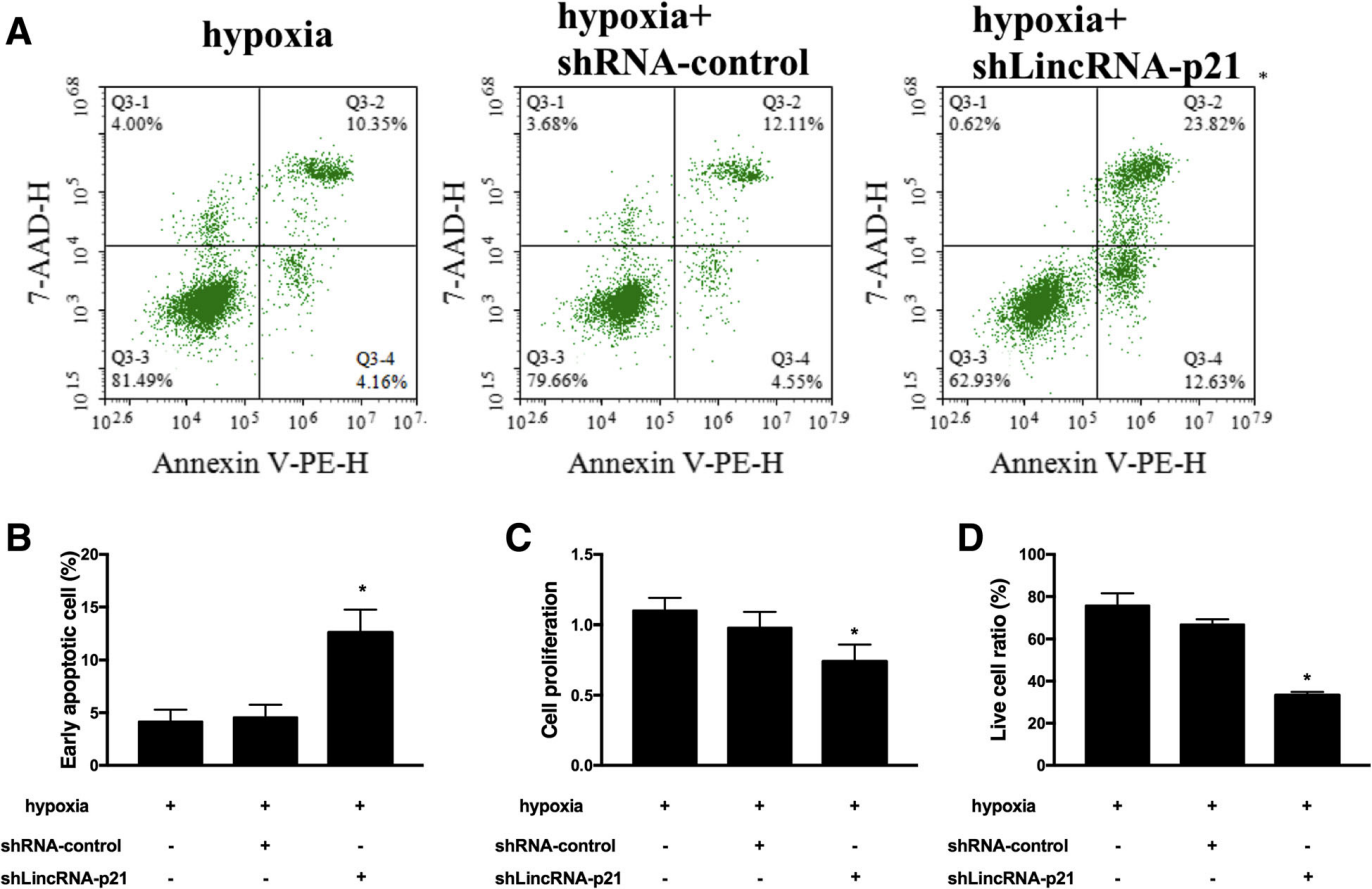
The effect of knock down of LincRNA-p21 on hypoxic preconditioning-induced MSC survival. To investigate the role of LincRNA-p21 on hypoxic preconditioning-induced MSC survival, we introduced shLincRNA-p21 and used hypoxic preconditioning (1% O_2_)-induced MSCs proliferation and apoptosis at 6 h, which were respectively tested by CCK8, trypan blue dye, and Annexin V-PE/7-AAD-stained flow cytometry method. **a** The effect of knock down of LincRNA-p21 on hypoxic preconditioning-induced MSC apoptosis with Annexin V-PE/7-AAD stained flow cytometry method at 6 h. **b** Early apoptotic MSC with knock down of LincRNA-p21 under hypoxic preconditioning at 6 h. **c** MSC proliferation with knock down of LincRNA-p21 under hypoxic preconditioning tested by CCK8 method at 6 h. **d** MSC proliferation with knock down of LincRNA-p21 under hypoxic preconditioning tested by trypan blue dye at 24 h. Results are mean ± SD (*n* = 3). *p < 0.05 vs. group hypoxia (+) shRNA-control (−) shLincRNA-p21 (−)

**Fig. 7:**
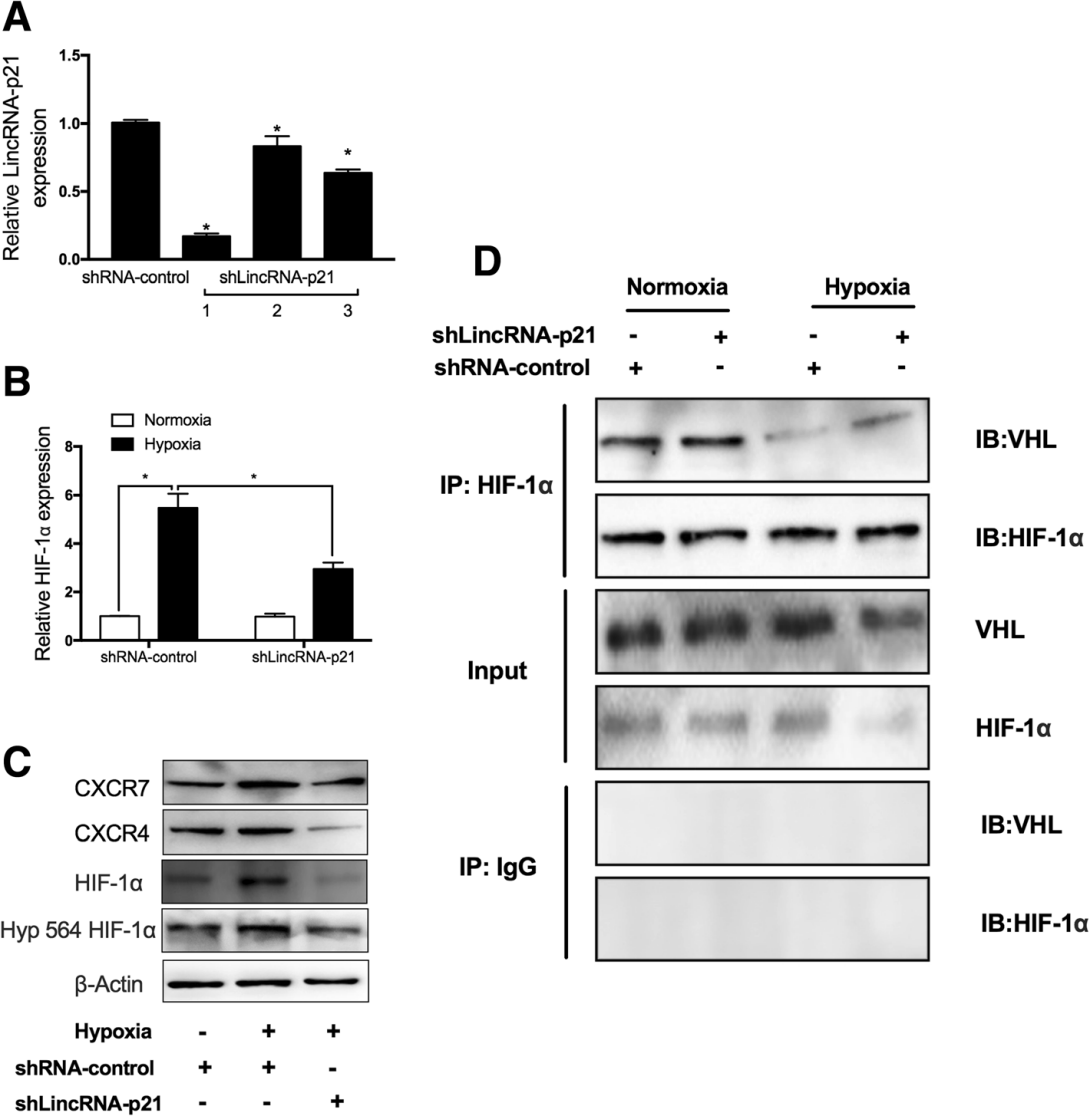
The effect of knock down of MSC LincRNA-p21 on HIF-1α and CXCR4/7 under hypoxic preconditioning. To research the role of MSC LincRNA-p21 on HIF-1α and CXCR4/7 under hypoxic preconditioning, we used shLincRNA-p21 to test signaling change by RT-PCR, WB analysis, and co-immunoprecipitation method to test signaling change under hypoxic preconditioning (1% O_2_) at 24 h. **a** Three different knock-down ways of LincRNA-p21 on MSC under hypoxic preconditioning. **b** The effect of knock down of MSC LincRNA-p21 on HIF-1α mRNA relative expression under hypoxic preconditioning. **c** The effect of knock down of MSC LincRNA-p21 on HIF-1α, Hyp564 HIF-1α, and CXCR4/7 protein expression under hypoxic preconditioning. **d** The effect of knock down of MSC LincRNA-p21 on interaction between HIF-1α and VHL protein. Results are mean ± SD (*n* = 3). *p < 0.05 vs. group shRNA-control or comparison

Since upregulation of HIF-1α and CXCR4/7 pathway by hypoxic preconditioning contributing to MSC migration and survival, the role of LincRNA-p21 on HIF-1α-mediated CXCR4/7 were further investigated. We used RNA interference to evaluate LincRNA-p21 effects on MSCs. We next used three different LincRNA-p21-targeting shRNAs to knock down LincRNA-p21 expression. The LincRNA-p21-1 had achieved the highest knock-down efficiency and was used in the subsequent study (Fig. 7a). MSC migration capacity was respectively researched by 24-h scratch test and transwell migration assays. The figure showed that shLincRNA-p21 suppressed the MSC healing area (Fig. 5a, b) and migration cell numbers (Fig. 5c, d) in hypoxic preconditioning MSC. The results referred that LincRNA-p21 under hypoxic preconditioning raised MSC migration. Moreover, LincRNA-p21 was also investigated in MSC survival, CCK8 analysis, and trypan blue dye for MSC proliferation and Annexin V-PE/7-AAD-stained flow cytometry for MSC apoptosis were introduced. Under hypoxic preconditioning, shLincRNA-p21 raised MSC early apoptosis (Fig. 6a, b). On the contrary, cell proliferation was decreased by shLincRNA-p21 (Fig. 6c, d).

Consistent with these, PCR method showed that shLincRNA-p21 responded to lower HIF-1α level (Fig. 7b). In addition, WB analysis displayed that shLincRNA-p21 blocked the upregulation of HIF-1α, Hyp564 HIF-1α, and CXCR4/7 under hypoxic preconditioning (Fig. 7c). To further investigate the relationship between LincRNA-p21 and HIF-1α, we applied co-immunoprecipitation method to observe HIF-1α and associated VHL protein. Knock down of LincRNA-p21 enhanced the HIF-1α-VHL interactions under hypoxia (Fig. 7d). Together, LincRNA-p21 contributed to HIF-1α-mediated expression of CXCR4/7 in MSC migration capacity and survival, and hypoxia-induced LincRNA-p21 stabilized HIF-1α- VHL interactions.

## Discussion

MSC target homing to injured lung tissue is an important process for MSC protective capacity and low efficiency in MSC homing to injured lung tissue remains to be puzzled. Thus, enhanced MSC homing capacity and survival are critical for MSC protective ability. Our study suggested that hypoxic preconditioning MSC with higher expression of LincRNA-p21 which could enhance MSC migration capacity and survival via HIF-1α/CXCR4 and CXCR7 pathway.

Hypoxic preconditioning has been recently reported to be involved in cell homing and migration to ischemic tissue [32]. Naderi-Meshkin et al. had reviewed various strategies to improve homing of MSCs [33]. Hypoxic preconditioning MSC is regarded to be extremely protective approach against subsequent lethal hypoxia damage [9, 10]. Hypoxic preconditioning refers to the brief hypoxic stimulus viscera in advance, making the viscera of subsequent hypoxia tolerance for a long time. Hypoxic preconditioning fully mobilizes the body’s protective reaction and inhibits traumatic reaction, and it is the most potent endogenous protective mechanism so far. In our study, we introduced scratch test and transwell migration assays to investigate the hypoxic preconditioning on MSC migration. The results showed that hypoxic preconditioning promoted MSC healing area and cellular migration numbers. These revealed that hypoxic preconditioning triggered MSC migration. We made further investigation to explore the effect of hypoxic preconditioning to MSC survival. Our data revealed that hypoxic preconditioning improved MSC survival by increasing cell viability and attenuating apoptosis. The results demonstrated that hypoxic stimulation had the positive effects of MSC migration and survival.

To explore mechanism of hypoxia preconditioning enhanced MSC migration and survival, we examined the expression of LincRNA-p21, HIF-1α, Hyp564 HIF-1α, CXCR4, and CXCR7 in MSCs after hypoxia preconditioning. HIF-1 is a key mediator of hypoxic response complex [34], consisting of HIF-1α (and subsequently HIF-2a and HIF-3a) as the O2-responsive subunit and HIF-1b as the constitutively expressed subunit [35]. HIF-1 regulates transcription of variety gene types in hypoxic conditions, including angiogenesis, erythropoiesis, cell cycle, metabolism, and apoptosis. Upon normoxic conditions, HIF-1α can be rapidly degraded by ubiquitin/proteasome pathways. Proline hydroxylation occurred in HIF-1α (Pro564 and Pro402), which resulted in conformational changes due to the binding of HIF-1α and von Hippel-Lindau (VHL) protein. This was followed by ubiquitination and proteasome degradation. However, hypoxia can inhibit HIF-1α degradation and lead to its stability [36, 37]. The protein encoded by VHL gene includes elongin B, elongin C, and cullin-2 and possesses ubiquitin ligase E3 activity, and it is a component of the protein complex. This complex plays roles in the ubiquitination and degradation of HIF. Studies showed that therapeutic effects were improved on hypoxic treatment due to HIF-1α stability in hypoxic cells [32, 38]. LincRNA-p21 was first reported to be a direct transcriptional target of p53 and to mediate p53-dependent apoptosis [39] and then has an important role in regulating p53-dependent cell cycle arrest in doxorubicin-treated mouse embryo fibroblasts [40]. Moreover, LincRNA-p21 transcripts were shown to increase in livers of mice treated with the carcinogen furan [41]. In recent study, LincRNA-p21 was discovered in increasing the available HIF-1α and regulation of angiogenesis and the Warburg effect [27–31]. Fan Yang et al. showed that LincRNA-p21 could be induced by HIF-1α under hypoxic conditions and contribute to disrupt the HIF1A-VHL interaction [27]. Meanwhile, other study [32] showed that HIF-1 was key regulators of cell response to hypoxia and could activate gene transcription of cell homing in anoxic conditions, which related with (chemokine stromal-derived factor-1) SDF-1/CXCR4. SDF-1/CXCR4 axis is considered to be key link in the process of stem cell homing. CXCR4/7 are receptors of chemokine stromal-derived factor-1 (SDF-1). The quantity expression of lung SDF-1 and CXCR4 was increased in bleomycin injured lung model and others [42–44]. Staller et al. [45] reported that a mechanism between VHL and CXCR4 activation during tumor cell evolution and the tendency to home to selected organs. In our study, MSCs were cultured under hypoxia environment (1% O2). MSCs induced by hypoxic preconditioning evoked an increase in expression of LincRNA-p21, HIF-1α, and CXCR4/7(SDF-1receptors). Next, we knocked down LincRNA-p21 of MSC and introduced HIF-1α inhibitor KC7F2 to explore mechanisms. Blockade of LincRNA-p21 by shRNA and HIF-1α inhibitor KC7F2 abrogated unregulation of hypoxic preconditioning induced CXCR4/7 in MSCs and cell migration and survival. Furthermore, co-immunoprecipitation assay revealed that hypoxic preconditioning isolated VHL and HIF-1α protein by increasing HIF-1α expression. These revealed that LincRNA-p21 and HIF-1α took part in MSC enhanced homing capacity and survival under hypoxic preconditioning. LincRNA-p21 stabilized HIF-1α protein via VHL under hypoxic preconditioning.

There are some limitations in our study. Our study only focuses on the effects of less than 24-h hypoxic preconditioning on MSCs; longer time effects were not certain. Moreover, it is just a cell experiment; more in vivo study should be investigated in following research to explore MSC homing capacity to lung injury.

## Conclusions

Here, we have shown that hypoxic preconditioning was identified as a promoting factor of MSC migration capacity and survival. LincRNA-p21 promotes MSC migration capacity via HIF-1α and CXCR4/7 through hypoxic preconditioning in vitro. Further animal experiment should be considered to explore MSC homing capacity to lung injury.

## Abbreviations

ARDS: Acute respiratory distress syndrome
CCK-8: Cell Counting Kit-8
CT: Threshold cycle
ELISA: Enzyme-linked immunosorbent assay
HIF-1α: Hypoxia-inducible factor
LncRNAs: Long non-coding RNAs
MSCs: Mesenchymal stem cells
RT-qPCR: Real-time quantitative-PCR
WB: Western blot
